# Image-Based Cardiac Diagnosis With Machine Learning: A Review

**DOI:** 10.3389/fcvm.2020.00001

**Published:** 2020-01-24

**Authors:** Carlos Martin-Isla, Victor M. Campello, Cristian Izquierdo, Zahra Raisi-Estabragh, Bettina Baeßler, Steffen E. Petersen, Karim Lekadir

**Affiliations:** ^1^Departament de Matemàtiques & Informàtica, Universitat de Barcelona, Barcelona, Spain; ^2^Barts Heart Centre, Barts Health NHS Trust, London, United Kingdom; ^3^William Harvey Research Institute, Queen Mary University of London, London, United Kingdom; ^4^Department of Diagnostic & Interventional Radiology, University Hospital Zurich, Zurich, Switzerland

**Keywords:** cardiovascular disease, automated diagnosis, cardiac imaging, artificial intelligence, machine learning, deep learning, radiomics

## Abstract

Cardiac imaging plays an important role in the diagnosis of cardiovascular disease (CVD). Until now, its role has been limited to visual and quantitative assessment of cardiac structure and function. However, with the advent of big data and machine learning, new opportunities are emerging to build artificial intelligence tools that will directly assist the clinician in the diagnosis of CVDs. This paper presents a thorough review of recent works in this field and provide the reader with a detailed presentation of the machine learning methods that can be further exploited to enable more automated, precise and early diagnosis of most CVDs.

## 1. Introduction

**Figure 1:**
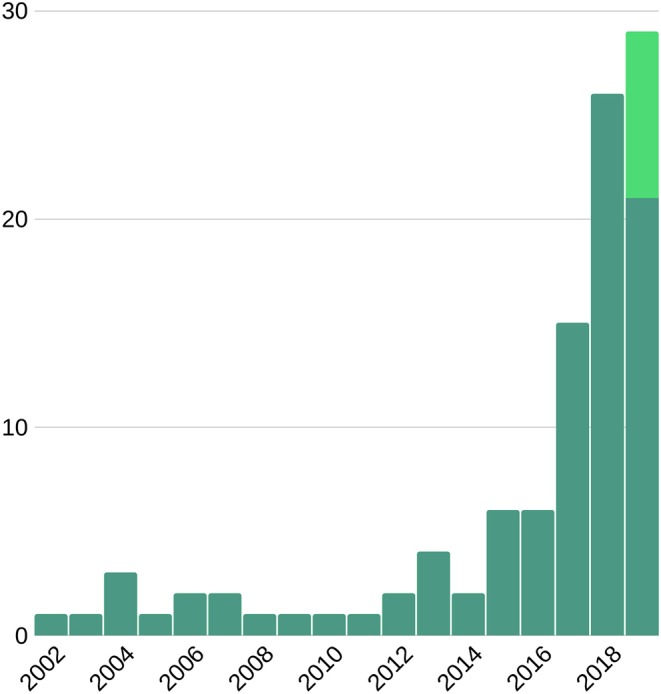
Number of publications on machine learning and cardiac imaging per year. This suggests an upward trend for future research. Light green bar represents the expected number of publications to be published late 2019.

Despite significant advances in diagnosis and treatment, cardiovascular disease (CVD) remains the most common cause of morbidity and mortality worldwide, accounting for approximately one third of annual deaths (1, 2). Early and accurate diagnosis is key to improving CVD outcomes. Cardiovascular imaging has a pivotal role in diagnostic decision making. Current image analysis techniques are mostly reliant on qualitative visual assessment of images and crude quantitative measures of cardiac structure and function. In order to optimize the diagnostic value 5 of cardiac imaging, there is need for more advanced image analysis techniques that allow deeper quantification of imaging phenotypes. In recent years, the development of big data and availability of high computational power have driven exponential advancement of artificial intelligence (AI) technologies in medical imaging (Figure 1). Machine learning (ML) approaches to image-based diagnosis rely on algorithms/models that *learn* from past clinical examples through identification of hidden and complex imaging patterns. Existing work already demonstrates the incremental value of image-based cardiovascular diagnosis with ML for a number of important conditions such as coronary artery disease (CAD) and heart failure (HF). The superior diagnostic performance of AI image analysis has the potential to substantially alleviate the burden of cardiovascular disease through facilitation of faster and more accurate diagnostic decision making.

In this paper we describe the main ML techniques and the procedures required to successfully design, implement, and validate new ML tools for image-based diagnosis. We also present a comprehensive review of existing literature pertaining to applications of ML for image-based diagnosis of CVD.

## 2. Overview of Pipeline for Image-Based Machine Learning Diagnosis

**Figure 2:**
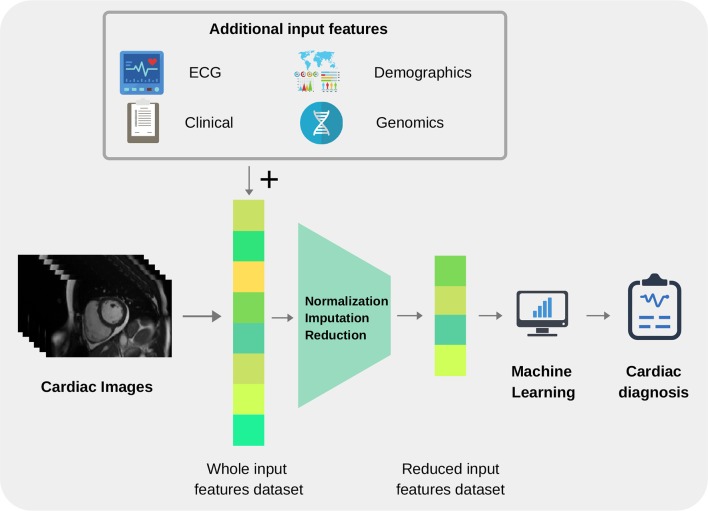
Pipeline for building image-based machine learning models.

The overall pipeline to build ML tools for image-based cardiac diagnosis is schematically described in the following section, as well as in Figure 2. In short, it requires (1) input imaging datasets from which suitable imaging predictors can be extracted, (2) accurate output diagnosis labels, and (3) a suitable ML technique that is typically chosen and optimized depending on the application to predict the cardiac diagnosis (output) based on the imaging predictors (input). Additional non-imaging predictors (e.g., electrocardiogram data, genetic data, sex, or age) are often integrated into the ML model and typically improve model performance.

In this section, we will first discuss the input and output variables in more detail, before introducing common used ML techniques and their applications.

### 2.1. Data, Input and Output Variables

#### 2.1.1. Sources of Cardiovascular Imaging Data

Robust ML models are reliant on the availability of sufficient and accurate data. Thus, data preparation is an important pre-requisite to derive that perform well on internal and external validation. Within cardiac imaging, there is increasing availability of quality sources of organized big data through various biobanks, bioresources, and registries. Available cohorts can be classified into population-based and clinical cohorts. Population cohorts such as the UK Biobank follow the health status of a representative sample of individuals from the general population and thus are particularly useful for risk stratification. In contrast, clinical cohorts, such as the Barts BioResource or the European cardiovascular magnetic resonance (EuroCMR) registry, are composed of clinical imaging from patients and therefore more suitable for building diagnostic tools. These datasets are an invaluable resource for the development and validation of ML diagnostic models (see Table 1 for examples of additional cardiac imaging datasets).

**Table 1:** Selection of cardiac imaging datasets available.

**Name**	**Country**	**Modality**	**Size**	**Year**
Framingham Heart Study	USA	Echo/MRI/CT	>5,000	1948
Study of Health in Pomerania	DE	MRI	>8,000	1997
The Tromso Study	NO	Echo	3,287	1999
Multi-Ethnic Study of Atherosclerosis	USA	MRI	2,450	2000
UK Biobank	UK	MRI	20,000	2006
HUNT Study	NO	Echo	1,296	2006
Defibrillators to Reduce Risk by MRI Evaluation	USA	MRI	450	2007
Barts BioResource	UK	Echo / MRI	>10,000	2007
European CMR Registry	EU	MRI	>27,000	2007
NEO Study	NL	MRI	1,205	2008
SunnyBrook Cardiac Data	USA	MRI	45	2009
Registry of Fast Myocardial Perfusion Imaging with next gen SPECT	USA	SPECT	>20,400	2009
The German National Cohort	DE	MRI	20,000	2011
Maastricht Study	NL	Echo / CT	3,451	2012
Canadian Alliance for Healthy Hearts and Minds	CA	MRI	9,700	2013
Challenge on Endocardial 3D Ultrasound Segmentation	FR	Echo	45	2014
Hamburg City Health Study	DE	MRI	>45,000	2016
Automated Cardiac Diagnosis Challenge Dataset	FR	MRI	150	2017
Cardiac Acquisitions for Multi-structure Ultrasound Segmentation	FR	Echo	500	2019

#### 2.1.2. Input Variables

**Figure 3:**
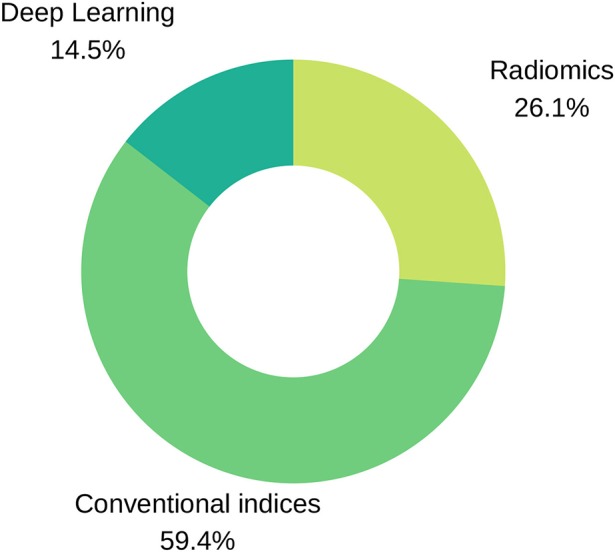
Input variables type distribution in reviewed literature. As seen in the pie chart, conventional indices are the predominant features for training ML models, followed by radiomics and deep learning techniques.

**Figure 4:**
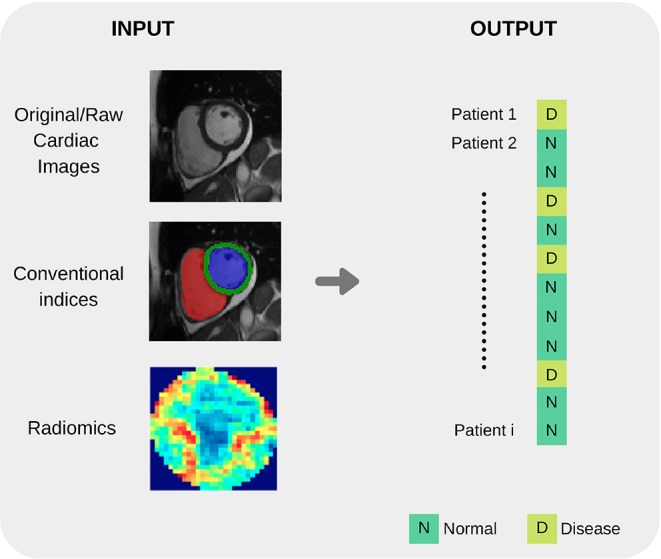
Summary of common input and output variables for image-based diagnosis ML algorithms. Different cardiac imaging input features such as raw data, conventional indices extracted from a ROI or radiomics (delineation of cardiac anatomy is required for the last two cases) and desired output. Both structures shape the most basic requirement for a ML cardiac imaging application, data.

Before an ML model can be built for image-based diagnosis estimation, it is necessary to suitably define the imaging inputs. Imaging inputs may be the raw imaging data (i.e., pixel intensities), conventional cardiac indices (and other transformed quantitative image parameters) or radiomics features extracted from the image. See Figures 3 and 4 for additional information about input variables.

##### 2.1.2.1. Conventional imaging indices

Conventional imaging indices include measures commonly used in routine clinical image analysis such as ventricular volumes in end diastole/systole and ventricular ejection fractions.

Estimation of these clinical indices requires prior contouring of the endocardial and epicardial boundaries of the relevant cardiac chambers. Deep learning approaches have been used to develop automated/semi-automated contouring tools for more efficient and reproducible segmentation of cardiac chambers.

Since manual delineation of these boundaries is tedious and subject to errors, many automatic or semi-automatic tools have been developed (see Table 2 for examples of existing tools). Note that recently, many deep learning (DL) based approaches have been published for accurate and robust segmentation of the cardiac boundaries with promising results, however this is beyond the scope of this review [more details on this, as well as a basic introduction to ML, in cardiac magnetic resonance imaging (MRI) can be found in recent work by (3)].

**Table 2:** Selection of cardiac structural and functional analysis softwares.

**Name**	**Producer**	**Modality**
CMRtools	Cardiovascular Imaging Solutions	MRI
suiteHEART	NeoSoft	MRI
CVI42	Circle Cardiovascular Imaging	MRI/CT
Medis Suite	Medis	MRI/CT
iNtuition	Terarecon	MRI/CT
Segment	Medviso	MRI/CT/SPECT
syngo.via	Siemens	MRI/CT/SPECT
IntelliSpace Portal	Philips	MRI/CT/echo
VevoLAB	Visualsonics	Echo
QLAB	Philips	Echo
TOMTEC	Philips	Echo

Some recent works will be listed to illustrate the use of conventional imaging indices as inputs for ML-based diagnosis models. In Khened et al. (4), an artificial neural network (ANN) was built to automatically diagnose several cardiac diseases such as hypertrophic cardiomyopathy (HCM), myocardial infarction (MI) and abnormal RV (ARV), by using as input LV and RV ejection fraction, RV and LV volume end-systole and end-diastole, myocardial mass, as well as the patient's height and weight. In Chen et al. (5), the authors integrated a set of 32 variables from clinical data, including ejection fraction, blood pressure, sex, age, as well as other conventional risk factors, to diagnose dilated cardiomyopathy (DCM). Juarez-Orozco et al. (6) merged ejection fractions at rest and stress with a pool of clinical parameters to predict ischemia and adverse cardiovascular events using ML.

Regarding motion, strain and single intensity analysis, in Mantilla et al. (7), global spatio-temporal image features are extracted to feed a support vector machine (SVM) classifier for LV wall motion assessment. Pairwise single intensity and variance regional differences in SPECT perfusion studies mimics the clinical procedure of qualitatively comparing stress and rest images in Bagher-Ebadian et al. (8). Contractility differences and multiscale wall motion assessment are performed by means of apparent flow in Moreno et al. (9) and Zheng et al. (10) where each feature describes an oriented velocity at a given position along the cardiac ROI.

##### 2.1.2.2. Radiomics features

Radiomics analysis is the process of converting digital images to minable data. Analysis of the data through application of various statistical and mathematical processes allows quantification of various shape and textural characteristics of the image, referred to as radiomics features (Table 3). Radiomics analysis quantifies more advanced and complex characteristics of the cardiac chambers than is visually perceptible. Similarly to clinical imaging indices, radiomics requires the delineation of the cardiac structures before the features can be extracted.

**Table 3:** Radiomics features overview.

**Type**	**Description**	**Examples**
Shape features	Describe geometric characteristics of the cardiac structures	Volume, surface area, sphericity, diameters, axis, surface to volume ratio, flatness
Intensity (First order)	Statistics on the intensity distributions within the region of interest (ROI)	Mean intensity, range, skewness (asymmetry) and entropy
Texture GLCM (Second order)	Quantifies the spatial relationship of the pixels in the ROI	Contrast, correlation
Texture GLSZM (Higher order)	Quantifies the number of connected voxels that share the same intensity level	Gray level non-uniformity, zone entropy
Texture GLRLM (Higher order)	Quantifies the gray level runs in the ROI	Run entropy, long run emphasis and short run emphasis
Texture NGTDM (Higher order)	Quantifies the difference between a gray value and the average gray value of its neighbors within a predefined distance	Busyness, strength
Texture GLDM (Higher order)	Quantifies the gray level dependencies in the ROI	Dependence non- uniformity, dependence entropy and dependence variance
Fractal dimension	Determines the ratio of change in detail to the change in scale	

Introduced in 2012 (11, 12), the radiomics paradigm was, for a long time, mostly exploited in oncology (13). Recently, a number of works have shown the promise of radiomics combined with ML for image-aided diagnosis of CVD. For instance, Cetin et al. (14) demonstrated that about 10 radiomics features integrated within an ML model are sufficient to discriminate between several major CVDs. More recently, researchers at Harvard University, Neisius et al. (15) have built an ML model with 6 radiomic features calculated from T1 mapping sequences to differentiate between hypertensive heart disease (HHD) and HCM.

##### 2.1.2.3. Raw imaging data

Whole raw images may also be used as the input for the ML model, without any pre-processing or calculation of hand-crafted input imaging features. About 10% of published reports rely on this type of modeling. In this case, the optimal features for predicting the cardiac diagnoses are self-learned automatically by the ML techniques based on the training sample, as opposed to *a priori* definition by the AI scientist.

For illustration, it is worth mentioning the work by Betancur et al. (16), an end-to-end DL model, estimating per-vessel CAD probability without any assumed subdivision of the input coronary territories from imaging data. The authors in Wolterink et al. (17) built a coronary artery calcification (CAC) detector, also based on DL trained on raw CT images. A similar DL model directly built from raw echo images was demonstrated in Lu et al. (18) to identify dilated cardiomyopathy cases. Also from raw echo images, the authors in Kusunose et al. (19) built a DL model for automatic detection of regional wall motion abnormalities.

#### 2.1.3. Output

ML algorithms may be developed using supervised or unsupervised learning methods. Supervised learning requires accurately labeled training examples. In the simplest form, the output is a binary variable which takes a value of 1 for a diseased individual and 0 for a control healthy subject. To obtain a robust ML model, it is recommended to use a balanced training sample, comprising a similar number of healthy and diseased subjects. Note that the binary classification can be easily extended to the multi-class case if several diseases or stages of disease are to be included in the ML model. Thus, supervised learning algorithms link the input variables to labeled outputs. Unsupervised learning is the training of algorithms without definition of the output. Through this technique, the ML algorithm groups the sample through recognition of inherent patterns within the data. In general, supervised learning outperforms unsupervised learning and so is the preferred method in situations where the ground truth is known. However, unsupervised learning has unique value for discovery of novel disease sub-types and patient stratification e.g., different pheno-groups of hypertensive heart disease or CAD.

### 2.2. Machine Learning Techniques

**Figure 5:**
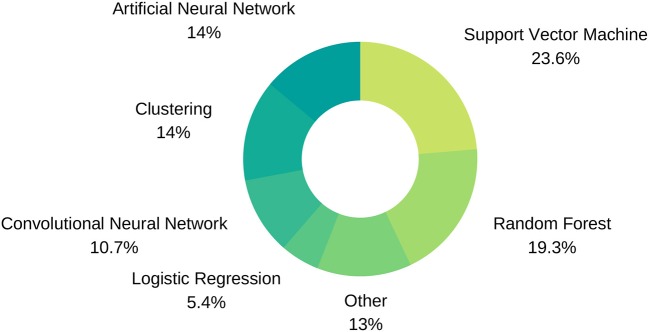
Machine Learning technique distribution.

ML, refers to the use of computer algorithms that have the capacity to learn to perform given tasks from example data without the need for explicitly programmed instructions, i.e., image-based cardiac diagnosis in our case. This field of AI uses advanced statistical techniques to extract predictive or discriminatory patterns from the training data in order to perform the most accurate predictions on new data. We present the most commonly used ML techniques in the field of cardiac imaging and diagnosis for a non-expert audience and discuss their benefits and drawbacks (see Table 4 and Figure 5 for additional information). A list of diagnostic applications for each method will be provided as examples.

**Table 4:** Overview of machine learning techniques.

**Technique**	**Description**	**Advantages**	**Disadvantages**
Logistic Regression	Extension of linear regression that outputs a binary classification	Simple and explainable; Does not require empirical parameter tuning nor input feature normalization	Not suitable for non- linear problems; Prone to overfitting
Support Vector Machine	Finds the optimal boundary between classes	Can handle different types of non-linear class separations; Does not require large training samples	Requires hyperparameter tuning and non-linear kernel selection; Not suitable for very large datasets
Random Forest	Generates a set of hierarchical decision queries over the input and output data	Automatically defines feature importances; Does not require input feature normalization	Prone to overfitting; Requires definition of depth and number of trees
Artificial Neural Network	Models complex classification tasks by propagating input data through a network of non-linear transformations	Generalizes well when trained on large training samples	Difficult to interpret; Requires prior selection of a network design (e.g., depth of the network); Requires large training data
Convolutional Neural Network	ANNs adjusted for the processing and classification of image data	Flexible design depending on the applications; Can learn the optimal features directly from the images	Same limitations as ANNs
Clustering	Finds subgroups within the input feature space in an unsupervised manner	Useful to discover subgroups when the groups labels are not unknown a priori; Simple and fast	Sensitive to initialization and scale; Difficult to estimate the number of subgroups

#### 2.2.1. Logistic Regression

**Figure 6:**
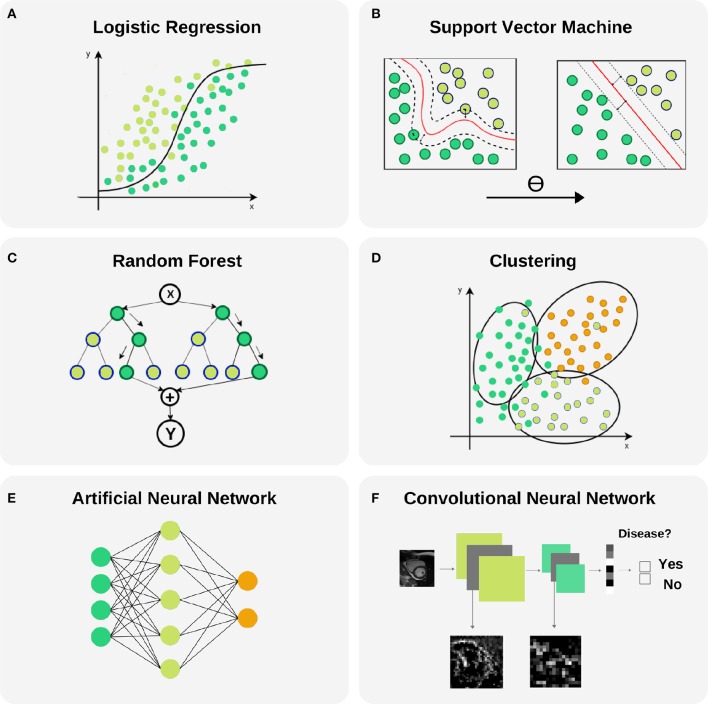
Selected machine learning techniques. **(A)** Logistic Regression is used to model the probability of a binary outcome. In the figure, Y axis represents the probability while X axis is the continuous input variable. Notice that small changes in X produce large variations of the final probability Y, mainly in the central part of the plot where the uncertainty of the model is larger. This model can be extended to a multi-class problems. **(B)** Support Vector Machine models are able to transform a non-linear boundary to a linear one using the kernel trick. During the training process, the distance between classes to the final selected boundary is maximized. **(C)** Random Forest is a technique that combines Decision Trees for reducing the uncertainty in the final prediction. It is based in a recursive binary splitting strategy where upper nodes are intended to be the most discriminative ones and subsequent branching is applied to less relevant variables. **(D)** Clustering is a technique with capability to find subgroups (clusters) along data. There are different cluster techniques, some need a prior number of clusters (kMeans), some of them can be used with output information (kNN), and others are fully unsupervised (meanShift). **(E)** Artificial neural networks are able to model complex non-linear relations between input variables and outcomes by propagating structured data (green nodes—input variables), e.g., radiomics, through hidden layers (blue nodes) to obtain an output (orange nodes). **(F)** Convolutional neural networks are the backbone of Deep Learning applications. They comprise input and output layers separated by multiple hidden layers. Their ability to hierarchically propagate imaging information and extract data-driven features implies automatic detection of relevant cardiac imaging biomarkers within the intermediate layers.

A Logistic Regression (LR) model is used to estimate the probability of a given output based on input variables in a continuous fashion, in contrast with a binary classifier. Final probabilities add up to one, so one obtains a stratification into all possible outcomes and the odds for each one. One property of this model is that a slight change in the input value may disproportionately impact the final probability prediction, as can be seen in Figure 6A. Additionally, the input vector dimension (number of predictor variables) must be kept low, as this can lead to costly model training processes and risks overfitting of the model to the training dataset with resultant poor generalisability of the model. Thus, when dealing with a large number of input variables, dimensionality reduction algorithms, such as principal component analysis (PCA) or linear discriminant analysis (LDA), are applied to reduce the number of predictors to those that are most informative. LR is a valuable model to be selected when different sources of data must be integrated in a binary classification task and low complexity is required.

In the literature, several works have applied LRs for their particular application. For example, Zheng et al. (10) applied a sequence of four LRs to classify patients according to cardiac pathologies by using shape features extracted from cine MRI per segment. Thus they obtained a simple and easily interpretable model with only three input features per classifier. In another example, Arsanjani et al. (20) used a combination of classifiers improved with a LR to diagnose obstructive CAD using SPECT images. Finally, a LR was also applied by Baeßler et al. (21) to diagnose acute or chronic heart failure-like myocarditis.

#### 2.2.2. Support Vector Machine (SVM)

Support vector machines (SVMs) are supervised ML models whereby the optimal linear or non-linear boundary segregating the data into two or more classes is identified, as can be seen in Figure 6B. Prior to application of SVMs, the function which will be used for segregating the data should be selected, the so called kernel function. The most used kernels are the linear function or the Gaussian function. The remaining parameters of the SVM model are chosen empirically by training a set of models and keeping the settings as for the model with the lowest error. Since this model is insensitive to non-discriminating dimensions, a dimension reduction could be applied to the input variables to ease the training and obtain a better generalization as for linear regression. One major drawback of SVM is that it becomes memory expensive when large amounts of data are processed. SVM is a good choice to identify non-linearity and sparsity in the input data : different kernels can be used to fit different distributions.

Amongst all ML methods presented in this review, SVM is one of the most frequently used techniques and some works find this model to obtain the best performance. For example, Conforti and Guido (22) presented a comparison of SVM models with different kernels (polynomial, Gaussian and Laplacian functions), the original 105 features and a feature selection of 25 as input for the early diagnosis of myocardial infarction. Similarly, Arsanjani et al. (23) and Ciecholewski (24) found that a SVM model outperformed previous algorithms used in the task of CAD identification by using data extracted from SPECT images. In the first example, a second degree polynomial was used as kernel while in the second, a Gaussian function showed better performance. A SVM was also the best model when predicting acute coronary syndrome for 228 patients using histological, ECG and echo qualitative features, as shown by Berikol et al. (25). As a final example, Borkar and Annadate (26) obtained a very good accuracy for discrimination of DCM and atrial septal defect (ASD) patients using radiomics features and a SVM using a Gaussian kernel function.

#### 2.2.3. Random Forest (RF)

This popular technique consists of a combination of decision trees (DTs) trained on different random samples of the training set, as can be seen in Figure 6C. Each DT is a set of rules based on the input features values optimized for accurately classifying all elements of the training set. DTs are nonlinear models and tend to have high variance. If the DT is grown very deep it can pick up irregularities in the training dataset and consequently problems with overfitting may be encountered. This problem is counteracted in a RF through training on different samples of the training dataset. In this way the variance is reduced as the number of DT used, lowering therefore the generalization error and becoming a powerful technique. The final prediction is obtained by selecting the mode (for classification problems) or the mean (for regression problems) of all predictions. Two parameters must be selected for these models: the number of DTs and the depth level for each DT (i.e., the number of decisions). However, one must bear in mind that whilst discriminatory power on training dataset is increased as DT increase in depth, this is often at the expense of losing generalization power. RFs are chosen in order to transform the problem into a set of hierarchical queries represented as DTs. However, RFs are not very resistant to noise.

In the literature, RF or DT have been used frequently and were selected as the best performing model in some works. For example, Moreno et al. (9) compared SVM and RF models in binary classification tasks with 2,964 input features for different cardiac pathologies, such as HF or HCM, using optical flow features in cardiac MRI, where the latter model obtained the best performance in most cases. In this case, each DT in the RF model had two depth levels for fast predictions in clinical practice. In another example, Wong et al. (27) a RF outperformed a SVM for infarction detection by means of regional intensity analysis and motion modeling. As a final example, a RF was also used by Baeßler et al. (28) to find the most discriminative features in texture analysis for T1-weighted cardiac MRI for HCM and normal patients classification.

#### 2.2.4. Cluster Analysis

Cluster analysis relates to the set of techniques that group together subjects in the form of data points according to similarity or proximity in the parametric space given by quantitative data extracted from input variables (image parameters and/or clinical information), as can be seen in Figure 6D. This technique is very useful for patient stratification, since patients with apparently similar pathology, according to existing image analysis techniques, may fall into previously unrecognized subsets which may inform understanding of disease pathophysiology and inform more effective targeted therapies. Some clustering techniques require definition of outcomes, which means that lay on the unsupervised learning ML group. However, in classification tasks a very common supervised clustering strategy is k-nearest neighbors (kNN) clustering, where k is the number of neighbor subjects to look at when finding subgroups. In this case, surrounding diagnosed subjects will determine the outcome for a new patient. Most of the reviewed literature in clustering uses kNN (29, 30).

Additional studies report the use of different cluster analysis for classification and/or discovery of cardiac pheno-groups. For example, Bruse et al. (31) used hierarchical clustering techniques to subdivide 60 patients into three groups, a healthy cohort and two associated with congenital heart disease by using shape features from cardiac MRI. Wojnarski et al. (32) also used a cluster analysis technique to group bicuspid aortic valve patients using CT data to find three phenotypes, and a RF was applied later to identify biomarker differences for these phenotypes using echo and clinical data.

#### 2.2.5. Artificial Neural Network (ANN)

ANNs are motivated by the structure and interactions of biological neural networks. These models propagate input data in a hierarchical fashion through internal nodes in different layers. Each input line has a corresponding weight that must be estimated and iteratively adjusted during the training process. The ANN adapts until the weights giving optimal model performance are identified (Figure 6E). A nonlinear function is applied in each node to the contribution from incoming connections for obtaining its value/activation (net input function). Weight optimization provides the model with great adaptability to complex boundaries separating classes because of the high non-linear combinations of features involved in such models. Moreover, the connections between layers in an ANN can be used to design different networks depending on the application. Some caveats are the lack of an underlying theory for deciding the amount of layers or nodes in each layer, that depends on each problem and the amount of training data, as well as the trend for these models to adapt to the training set due to the large difference between number of parameters/weights of the model and training samples. ANNs are the best choice when large amount of data is available.

In the literature, these techniques have been applied frequently. For example, Tsai et al. (33) used ANNs for detection of HCM and DCM patients using features extracted from echo. And more recently, two works by Nakajima et al. (34, 35), with the same SPECT dataset with 1,001 cases, used ANNs to assess CAD using features extracted from stress and rest images with good accuracy.

#### 2.2.6. Convolutional Neural Network (CNN)

CNNs are an extension of ANNs in which the value of a node in a given layer is affected by the spatial surrounding of a node in the previous layer through an operation called convolutional product. These models are specially designed for image processing, where spatial information for the nodes (pixels) is essential for the final prediction. The advantages and disadvantages are shared with ANNs. The main difference that make these models very popular nowadays is that images are provided as input without any feature extraction. These models are able to extract their own meaningful features for the final prediction, as illustrated in Figure 6F. Additional models exist for compressing images to a lower dimensional representation space such as the Variational Autoencoder (VAE) and Generative Adversarial Networks (GANs) where additional analysis can be carried out more easily (e.g., clustering or classification with a SVM model).

A balanced approach should be taken to defining the layers of a CNN; whilst a deeper network loses information from the original image with each new layer, a network with few layers could have problems extracting meaningful features for the final prediction. CNNs are widely used for analysis of images and their application to cardiac imaging is reported in numerous studies. Wolterink et al. (17) presented a framework where two cascading CNNs were able to detect CAC using cardiac computed tomography angiography (CTA) images. Their models had 8–13 convolutional layers that reduced 200 × 200 features (pixel intensities) to only 32. Zhang et al. (36) used a 13-layer CNN to diagnose HCM, cardiac amyloidosis and pulmonary artery hypertension from echo images of size 224x224, that were reduced to 4,096 features. Madani et al. (37) used a CNN model to predict left ventricular hypertrophy from echo images of size 120 × 160.

#### 2.2.7. Additional Steps

##### 2.2.7.1. Normalization

Due to the diverse nature of different information sources in cardiac medicine, a normalization step is often required prior to model crafting. In general, learning algorithms benefit from standardization of the data set, e.g., some algorithms as SVM will improve cardiovascular predictions if all numerical features are zero centered and have a variance of the same magnitude order. Furthermore, some non-linear transformations can prepare the selected features to create a model more robust to outliers. Some of the most common techniques are mentioned in Table 5.

**Table 5:** Common normalization techniques.

**Technique**	**Description**	**Advantages**
Mean/variance normalization	Centering to zero mean and unit variance	Avoid high variance features dominance
Range scaling	Mapping to a given interval	Robustness to small variances, preserve zero entries
Robust scaling	Mapping interval with	Robustness to outliers interquartile information
Image normalization	Brightness/contrast correction	Avoid variability in pixel intensity distribution

For illustration, Wong et al. (27) shows that feature normalization has a positive impact in the ML model performance. Moreover, categorical variables should be encoded using *Integer encoding*, that consist in referencing each possible categorical value with an integer, or *One-Hot encoding*, that considers each possible categorical value as a new binary variable.

##### 2.2.7.2. Dimensionality reduction and feature selection

Frequently, after extracting features from different sources such as demographic and clinical data, conventional indices and imaging parameters, one ends up with thousands of values defining a single patient. This information is later utilized during the training process of ML models, but the combination of a large number of input parameters with a limited number of samples (as usually happens in the medical field) can make the optimization problem expensive and may limit the generalization ability of our model. Thus, a dimensionality reduction algorithm is usually applied to the input data, such as principal component analysis (PCA) or linear discriminant analysis (LDA). Another proactive approach is feature selection. Such method will add sequentially the most discriminative features for the particular model instance being trained and dismiss redundant and non-informative ones.

For example, Tabassian et al. (29) aimed to analyze deformation curves of the LV in echocardiographic records of 120 patients. The strain curves obtained were reduced by means of PCA and the result was used to train a strain kNN model. The resultant accuracy was 0.87, significantly higher than the clinician's results, 0.7. Cetin et al. (38) identified HHD from healthy controls in 200 subjects with SVM and sequential forward feature selection. The predictive power of selected radiomics (AUC = 0.76) was substantially improved compared to conventional indices (AUC = 0.62).

#### 2.2.8. Validation

In order to prove the validity of ML applied to cardiac imaging, results must be analyzed from two perspectives: statistical validity, considering the reproducibility with different cohorts and correctness of statistical values obtained (i.e., metrics), and intra-validity, regarding the clinical and real implications of the algorithms on a daily basis (i.e., clinical effectiveness). This is a pairwise co-existence; none of the ML cardiac imaging algorithms will be applied in clinical routine if there is no agreement from both sides. The following sub-sections will describe how the metrics and the clinical effectiveness are considered.

A cohort is sorted in a very specific manner for ML purposes. For the validity of the algorithms, a whole cardiac imaging data set should be split into 3 different subgroups, called *training set, validation set*, and *testing set*, respectively. These groups are often selected in such way that subgroups share demographic distributions such as age or sex, in order to represent a real world scenario. Of course, a balanced distribution of control and pathologic subjects is also required. Once the ML model is trained and tested, different metrics are obtained to evaluate its performance.

*Accuracy* measures the percentage of the algorithm classifying the input data correctly. It is a simple measure used in multiple scientific scenarios if there is no class imbalance (i.e., one class represented by a higher number of individuals compared with the rest). One of the drawbacks of using accuracy as the metric is that there is a knowledge loss when measuring False Positive and False Negative observations. Therefore, *Specificity* (Sp) and *Sensitivity* (Se) are widely used for measuring the performance of the algorithm, this time taking into consideration a possible class imbalance. In order to assess the performance of an algorithm and to understand where there might be a miss-classification issue, a table report called *Confusion Matrix* is used. This specific table layout is typically used to describe the performance of a supervised learning model. Each row of the matrix represents the instances in a predicted class while each column represents the instances in an actual class (or vice versa). This way, a computer scientist can have a wider overview of the parameters that may be changed or which classes are down-performing the algorithm. From sensitivity, specificity and the confusion matrix we can extract a performance plot representation called the receiver operating (ROC) *curve*. It is created by plotting the true positive rate (TP rate) against the false positive rate (FP rate) at various threshold settings. In ML, the true-positive rate is also known as sensitivity, recall or probability of detection. ROC analysis is related in a direct and natural way to cost/benefit analysis of diagnostic decision making. The area under the ROC curve (AUC) is another metric used to measure algorithms' performance.

It is noticeable that AUC can be derived from decision boundaries obtained by ML models despite the fact that it is trained with discrete outputs. When a trained model is asked to make a prediction, a probability can be computed and used to generate a ROC analysis.

## 3. Diagnostic Applications—A Review of Literature

**Figure 7:**
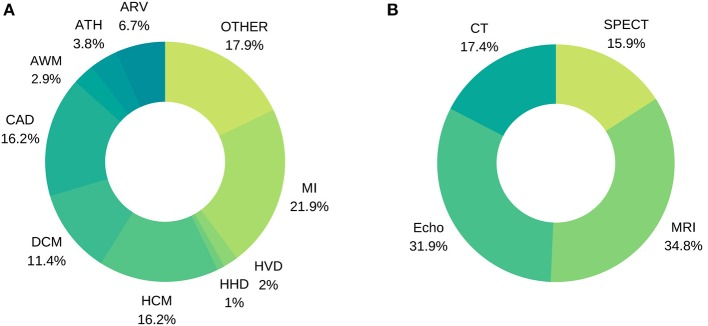
Distribution of image-based diagnostic application using machine learning **(A)** per disease, **(B)** per modality.

We conducted an organized, pre-defined literature search of two electronic databases (Google Scholar, Scopus). We included studies using a well-defined ML technique for cardiac image analysis using echocardiography, cardiac magnetic resonance, cardiac computed tomography, or single photon emission computed tomography (SPECT). Our search strategy comprised a series of title and whole text searches with search terms combined using Boolean operators. Search results were filtered by subject area, limiting to entries from Cardiology, Computer Science and Engineering fields. We review in detail various achievements in the diagnosis of a wide range of cardiac diseases using image-based ML methods. Statistics about the conducted literature review can be seen in Figure 7.

### 3.1. Myocardial Infarction

Accurate and timely identification of MI helps in guidance of treatment strategies and reduction in the time taken for further tests. While MI diagnostic assessment using imaging is prone to inter- and intra-observer variability and requires significant amount of time of experts, ML methods offer opportunities to simplify, speed up and quantify the diagnostic process in combination with conventional assessment. For example, Nakada et al. (39) demonstrated that MI diagnosis can be achieved in echo using quantitative motion features, avoiding the inter-observer human variability, as input for an ANN reaching an accuracy of 0.95. Later, Ungru et al. (40) validated these results in mice models by inducing MI in healthy specimens with a prediction accuracy of 0.91, comparing several ML techniques. The same level of accuracy was obtained in the first texture analysis work, by Agani et al. (41), with only 17 subjects and a clustering approach. This echocardiographic research was later extended with a full pool of texture features and 160 subjects by Sudarshan et al. (42). In this work, DT, ANN and SVM models were benchmarked, with the best accuracy obtained using ANN: 0.94 (Se = 0.91, Sp = 0.97). Vidya et al. (43) also performed an intensive texture analysis for 800 subjects, achieving an accuracy of 0.99 using a SVM. In their study, different pre-processing techniques were used to enhance the cardiac images.

Cardiac MRI has particular value in identification of MI. Since 2017, 13 studies were found integrating input variables from this imaging modality. Baeßler et al. (44) used late gadolinium enhancement MRI as a standard reference for non-enhanced MRI discrimination between chronic and subacute MI. Radiomic features in combination with a LR gave an AUC of 0.92 in a cohort of 180 patients. Similarly, segment viability can be detected on cine MRI using also radiomics, as suggested by Larroza et al. (45). This classification between viable, non-viable and remote segments yielded an AUC of 0.84. However, we believe that these encouraging results should be validated with a bigger cohort, and a well-balanced segment viability distribution. Recently, Zhang et al. (46) tried to detect MI from non-enhanced MRI images. 212 patients with chronic MI and 87 healthy control patients were used to train a three-stage DL pipeline. The per-segment AUC for detecting chronic MI was 0.94 (Sp = 0.99, Se = 0.9)

Two consecutive state-of-the-art texture analysis studies were conducted in cardiac CT: Mannil et al. (47) and Mannil et al. (48). The former underlines ML ability for detecting MI on non-contrast low radiation dose CT images on the basis of features invisible to the radiologists' eye, obtaining an AUC of 0.78. The latter study evaluates the impact of automatic classification methods using different iterative reconstruction (IR) strengths for contrast-enhancement images, reporting an accuracy of 0.94 (IR 3) and 0.97 (IR 5) for the ML model, while three independent readers achieved 0.73 (IR 5) on average. A summary of MI studies can be found in Table 6.

**Table 6:** Selected studies using image-based ML analysis for the diagnosis of Myocardial Infarction.

**Publication**	**Modality**	**Biomarker**	**ML technique**	**Diagnostic**	**Sample size**	**Performance**
(44)	MRI	Radiomics	LR	MI	180	ACC = 0.92
(46)	MRI	Conventional	ANN	MI	299	AUC = 0.94
(45)	MRI	Radiomics	SVM	MI	50	AUC = 0.84
(9)	MRI	Conventional	SVM/RF	MI/HCM	45	ACC = 0.94
(94)	MRI	Conventional	DT/CL/SVM	MI	200	ACC = 0.95
(95)	MRI	Conventional	PLS	MI	200	ACC = 0.98
(22)	Echo	Qualitative	SVM	MI	242	ACC = 0.97
(39)	Echo	Conventional	ANN	MI/AP	91	ACC = 0.95
(40)	Echo	Conventional	BN/DT/CL/SVM	MI	42	ACC = 0.91
(42)	Echo	Radiomics	DT/ANN/SVM	MI	160	ACC = 0.94
(41)	Echo	Radiomics	CL	MI	17	ACC = 0.91
(43)	Echo	Radiomics	SVM	MI	800	ACC = 0.99
(29)	Echo	Conventional	CL	MI	120	ACC = 0.87
(47)	CT	Radiomics	RF/CL/ANN	MI	87	ACC = 0.78
(48)	CT	Radiomics	DT	MI	30	ACC = 0.97
(27)	CT	Conventional	SVM/RF	MI	170	ACC = 0.85
(96)	SPECT	Conventional	BN	MI/CAD	728	ACC = 0.78

### 3.2. Cardiomyopathies

Cardiomyopathy is a broad term describing various heart muscle disorders, a first level of subclassification is into ischaemic and non-ischaemic cardiomyopathies. This heterogenous group of disorders have many causes, signs and symptoms, and require different treatments. The challenge of distinguishing different cardiomyopathies is illustrated by the fact that many of them can be associated with diverse manifestations. Each disease entity is associated with a typical imaging phenotypes. Whilst in routine image analysis, it is not always possible to discriminate individual cardiomyopathies, this may be improved with the more granular and quantitative approach to image analysis in ML models. These premises makes ML-based imaging diagnosis a perfect tool for computer aided analysis of heterogeneous cardiomyopathies. For example, Gopalakrishnan et al. (49) used a set of conventional indices from a pediatric cardiac MRI cohort of 83 subjects to characterize five different cardiomyopathies. In this study, a DT (AUC = 0.79) was compared with other ML methods (AUC = 0.73–0.77). Physiological vs. pathological patterns of HCM remodeling were characterized by Narula et al. (50) using an ensemble of models with conventional indices from 2D echo as input (Se = 0.96, Sp = 0.77).

In 2017, a relevant challenge was organized by Bernard et al. (51). The Automated Cardiac Diagnosis Challenge (ACDC) aimed to evaluate the performance of different automatic methods for the classification of 150 subjects into 5 categories (healthy, HCM, DCM, ARV and MI) as provided by clinical experts. Several approaches were proposed for this problem. Khened et al. (4) and Wolterink et al. (52) used a set of conventional indices extracted from their own automatic delineations as input for a RF to obtain an accuracy of 0.96 and 0.86 on the test set, respectively. Isensee et al. (53) also used a RF and their own segmentation scheme to classify cardiac cycle dynamic features, with an accuracy of 0.92. From this study, the benefit of the addition of temporal analysis is remarkable and provides a strong argument to be exploited further in future cine MRI studies. Cetin et al. (14) used SVM to classify a complete pool of radiomic features from manual segmentation, obtaining also an accuracy of 0.92. Additional research has been done later using the same dataset. Snaauw et al. (54) proposed a novel approach, using CNN bottleneck representations to discriminate between the five categories, obtaining a modest accuracy of 0.78. Another interesting approach was taken by Biffi et al. (55). Their VAE architecture was trained with two multi-center cohorts of 537 and 200 patients and tested on their own dataset and on the ACDC dataset, obtaining an accuracy of 1.0 and 0.9, respectively.

Later, Puyol-Antón et al. (56) combined MRI and echo data and per-segment motion analysis to diagnose DCM by means of LDA, achieving an accuracy of 0.94 (Sp = 0.96, Se = 0.93). Recently, Neisius et al. presented two complementary works approaching HCM and HHD diagnosis from two different perspectives, Neisius et al. (15, 57). In the first work, a complete strain analysis and a LR achieved an accuracy of 0.67 (Sp = 0.64, Se = 0.68). The second one applied an exhaustive texture analysis for T1 mapping. A selection of 6 radiomic texture features and a linear SVM model showed an improved accuracy of 0.86 (Sp = 0.91, Se = 0.77). A summary of cardiomyopathy studies can be found in Table 7.

**Table 7:** Selected studies using image-based ML analysis for diagnosis of various cardiomyopathies.

**Publication**	**Modality**	**Biomarker**	**ML technique**	**Diagnostic**	**Sample size**	**Performance**
(49)	MRI	Conventional	BN	HCM/DCM/ARV/MYO	83	AUC = 0.79
(28)	MRI	Radiomics	RF/LR	HCM	62	AUC = 0.95
(52)	MRI	Conventional	RF	MI/HCM/DCM/ARV	100	ACC = 0.86
(14)	MRI	Radiomics	SVM	MI/HCM/DCM/ARV	100	ACC = 0.92
(53)	MRI	Conventional	RF	MI/HCM/DCM/ARV	100	ACC = 0.92
(4)	MRI	Conventional	RF	MI/HCM/DCM/ARV	100	ACC = 0.96
(55)	MRI	Deep Learning	VAE	HCM	737	ACC = 1.00
(10)	MRI	Conventional	LR	MI/HCM/DCM/ARV	100	ACC = 0.94
(57)	MRI	Conventional	LR	HHD/HCM	224	ACC = 0.67
(15)	MRI	Radiomics	SVM	HHD/HCM	224	ACC = 0.86
(54)	MRI	Deep Learning	CNN	MI/HCM/DCM/ARV	100	ACC = 0.78
(9)	MRI	Conventional	SVM/RF	MI/HCM	45	ACC = 0.94
(31)	MRI	Conventional	CL	CHD	60	ACC = 0.89
(50)	Echo	Conventional	SVM/RF/ANN	HCM/ATHCM	139	ACC = 0.91
(33)	Echo	Radiomics	ANN/GA	HCM/DCM	90	ACC = 0.95
(18)	Echo	Deep Learning	CNN	HCM/DCM	927	AUC = 0.84
(56)	Echo/MRI	Conventional	SVM	DCM	69	ACC = 0.94
(26)	Echo	Radiomics	SVM	DCM/ASD	439	ACC = 0.98
(37)	Echo	Deep Learning	CNN/GAN	HCM	772	ACC = 0.92
(36)	Echo	Deep Learning	CNN	HCM/CA/PH	14,035	AUC = 0.93

### 3.3. Coronary Artery Disease

Non-invasive imaging assessment for detection of CAD has a great potential impact on clinical practice. If ischemia can be discarded with a high probability, invasive coronary angiography (ICA) may be avoided. Advanced ML image analysis techniques can improve the diagnostic accuracy of myocardial ischemia and through this improve CAD management and reduce unnecessary downstream testing.

A very first approach dating from 1999 showed promising results. Considering ICA as reference standard, Kukar et al. (58) used scintigraphy, ECG and data on symptoms from 327 patients to detect CAD. Different ML models and feature selections were tested and in some cases the ML model outperformed clinicians in accuracy (0.92 vs. 0.91, respectively), but not in sensitivity. An exhaustive approach by Kurgan et al. (59) sets the base for a semi-automated diagnosis pipeline in perfusion SPECT. In their work, a pseudo-DT was crafted from intensity-based features, for 267 subjects, achieving an overall accuracy of 0.8. Another similar work was conducted in perfusion SPECT (*n* = 115) and Equilibrium Radionuclide Angiocardiography (*n* = 58) by Bagher-Ebadian et al. (8). Using ICA as ground truth for both studies, CAD was assessed using mean and variance intensity features extracted from stress and rest studies in anterior, left anterior oblique and left lateral projections, obtaining accuracies of 0.77 and 0.73 with an ANN. A similar methodology was covered in detail by Guner et al. (60). A cohort of 308 patients with clinical coronary CTA assessment was utilized to train an ensemble of ANNs for CAD discrimination. A combination of demographic information and frequency, phase and brightness features provided as input variables resulted in model accuracy of 0.74, outperforming some of the non-expert clinicians. The results revealed that single-vessel CAD was more difficult to identify. Recently, complementary work by Shibutani et al. (61), including per-segment analysis, was performed on 21 patients who underwent perfusion SPECT. A total of 109 abnormal regions were examined and an ANN achieved better results than two independent observers for stress defect and ischemia detection, with respect to ICA as gold standard.

Alternatively, resting CT can be used for CAD diagnosis without additional contrast injection for stress imaging. Han et al. (62) used 3 quantitative features and the 17-segment model to obtain 51 input variables for training a gradient boosting algorithm, a ML technique that builds an ensemble of classifiers to improve the final accuracy. Invasive angiography and FFR were used as gold standard. This study based on a 252 patients' cohort from 5 countries and 17 centers, obtained an AUC of 0.75. Another state-of-the-art approach using cardiac CT, by Coenen et al. (63), showed that improved reclassification of non-significant stenosis is possible with ML-based image analysis. Three hundred and fifty-one patients, including 525 vessels with invasive FFR comparison were included in this study. A set of 28 anatomical features were computed from semi-automatic 3D CT reconstructions. On a per-vessel basis, diagnostic accuracy improved from 0.58 (CTA) to 0.78 (ML model). The per-patient accuracy improved from 0.71 to 0.85. A summary of CAD studies can be found in Table 8.

**Table 8:** Selected studies using image-based ML analysis for diagnosis of coronary artery disease.

**Publication**	**Modality**	**Biomarker**	**ML technique**	**Diagnostic**	**Sample size**	**Performance**
(20)	SPECT	Conventional	LB	CAD	1,181	AUC = 0.94
(16)	SPECT	Deep Learning	CNN	CAD	1,160	AUC = 0.81
(34)	SPECT	Conventional	ANN	CAD	1,365	AUC = 0.75
(35)	SPECT	Conventional	ANN	CAD	106	AUC = 0.96
(60)	SPECT	Conventional	ANN	CAD	65	AUC = 0.74
(97)	SPECT	Conventional	DT/GA	CAD	267	ACC = 0.83
(24)	SPECT	Qualitative	SVM	CAD	267	ACC = 0.92
(8)	SPECT	Deep Learning	ANN/CL	CAD	173	AUC = 0.80
(61)	SPECT	Conventional	ANN	CAD	109	AUC = 0.88
(6)	PET	Conventional	N/A	CAD/MACE	1,234	AUC = 0.72
(63)	CT	Conventional	N/A	CAD	352	AUC = 0.84
(62)	CT	Conventional	GBRT	CAD	252	AUC = 0.75
(58)	echo/SCI	Qualitative	ANN	CAD	327	ACC = 0.80
(98)	echo	Radiomics	SVM	CAD	61	AUC = 0.88
(25)	echo	Qualitative	SVM	CAD	228	ACC = 0.99

### 3.4. Atherosclerosis

Atherosclerosis is a strong and independent predictor of cardiovascular events. Plaque is often scored manually by experts, which leads to an increase in workload, is prone to false positives and to inter-observer variability regarding CAC detection. Hence, the ability to quickly and reliably quantify calcification using ML models provides additive value to clinical risk scoring tools and will enable superior prognostication of individuals. To overcome these issues and bring robustness to such procedures, intensive cardiac imaging feature extraction may be utilized.

Išgum et al. (30) designed an automated method for detection of aortic calcification, an indicator of established atherosclerotic disease, based on shape and intensity features. Forty abdominal scans contained a total of 249 CAC determined by a human observer. The method detected 209 CAC (Se = 0.84) at the expense of 1.0 false-positive object per scan on average, while the presence of contrast increased the number of incorrect classifications. This work was complemented by Išgum et al. (64), analysing cardiac CT with a more sophisticated feature set to obtain a final accuracy of 0.74 for CAC detection. Feature selection showed that no shape features were included in the classification stage, highlighting the discriminating power of texture analysis in CT.

Wolterink et al. (65) used cardiac CT scans thresholded at 130 Hounsfield units and a connected-component analysis to obtain candidate regions in the coronary arteries for 164 subjects with expert annotations. Their texture analysis was similar to Išgum et al. (64), and the resulting accuracy with DTs was 0.86 for risk stratification. This work also introduced a guided review where the most uncertain CAC were manually inspected again, increasing the overall accuracy up to 0.92. Later, a large radiomic pool of 4,440 features was extracted from a group of 60 subjects with Napkin Ring Sign (NRS) and non-NRS plaques with similar degree of manually segmented CAC by Kolossváry et al. (66). This research unveils the value of radiomics to find discriminative features: almost half of them reached an AUC of 0.8, short- and long-run low gray-level emphasis and surface ratio of high attenuation voxels had the highest AUC values (0.92 and 0.89, respectively). Finally, in a recent work, Zreik et al. (67) used recurrent CNNs in multi-planar reformatted coronary CTA images previously annotated by an expert, achieving accuracies of 0.77 and 0.8 for plaque and stenosis characterization, respectively. A summary of ATH studies can be found in Table 9.

**Table 9:** Selected studies using image-based ML analysis for diagnosis of aortic and coronary atherosclerosis.

**Publication**	**Modality**	**Biomarker**	**ML technique**	**Diagnostic**	**Sample size**	**Performance**
(66)	CT	Radiomics	N/A	ATH	60	AUC = 0.91
(67)	CT	Deep Learning	CNN	ATH	163	ACC = 0.80
(65)	CT	Radiomics	DT	ATH	164	ACC = 0.86
(17)	CT	Deep Learning	CNN	ATH	250	ACC = 0.72
(64)	CT	Conventional	CL	ATH	615	ACC = 0.74
(30)	CT	Conventional	CL	ATH	249	ACC = 0.83

### 3.5. Valvular Heart Disease

Heart valve disease is an increasingly common pathology of the cardiovascular system and an increasing number of patients are expected to require heart valve replacement. Such diverse group of disorders can benefit from cardiac imaging ML integration through early diagnosis, treatment or surgery planning. For instance, Elalfi et al. (68) used imaging preprocessing techniques (Gaussian and Gabor filtering) and intensity and texture features to generate an ANN model with 120 echo images. These images were organized in 8 types of valvular diseases. The obtained accuracy was high at 0.93. This is encouraging particularly considering the diversity of outcomes.

A similar approach was addressed for mitral regurgitation (MR) severity estimation using echo videos. Moghaddasi et al. (69) took advantage of binary patters as image descriptors which include details from different viewpoints of the heart. kNN and SVM models were trained with 102 patients divided in four groups: mild MR (*n* = 34), moderate MR (*n* = 32), severe MR (*n* = 36), and control (*n* = 37). SVM obtained the best accuracy, 0.99. Another interesting work mentioned in previous sections was conducted by Wojnarski et al. (32). A summary of HVD studies can be found in Table 10.

**Table 10:** Selected studies using image-based ML analysis for diagnosis of valvular heart disease.

**Publication**	**Modality**	**Biomarker**	**ML technique**	**Diagnostic**	**Sample size**	**Performance**
(68)	echo	Radiomics	ANN	HVD	120	ACC = 0.93
(69)	echo	Radiomics	SVM/CL	HVD	102	ACC = 0.99
(32)	CT	Conventional	CL	HVD	656	N/A

### 3.6. Heart Failure

Heart failure with preserved ejection fraction (HFpEF) is a heterogeneous group of disorders with variable treatment response and poor outcomes. There has been increasing interest in improved phenotyping of HFpEF to aid understanding of underlying disease mechanisms and also to guide treatments toward subtypes who may derive benefit. Given the heterogeneous nature of HFpEF, ML techniques are a very suitable tool for diagnosis and image phenotype stratification. Some of the reviewed studies in previous sections were also related to the characterization of heart failure (9, 70). Additional work in this field was presented by Shah et al. (71), that prospectively studied 397 HFpEF patients and performed detailed clinical, laboratory, electrocardiographic and echocardiographic phenotyping of the study participants. Clustering techniques were applied to divide the cohort into 3 pheno-groups. Phenomapping was helpful for improved classification and categorization of HFpEF patients and risk stratification by means of SVM, obtaining an AUC of 0.76. ML applied to HF phenogrouping is also used for prognostic tasks by Cikes et al. (72). A summary of HF studies can be found in Table 11.

**Table 11:** Selected studies using image-based ML analysis for diagnosis of heart failure.

**Publication**	**Modality**	**Biomarker**	**ML technique**	**Diagnostic**	**Sample size**	**Performance**
(9)	MRI	Conventional	SVM/RF	MI/HCM/HF	45	ACC = 0.77
(21)	MRI	Radiomics	LR	HF	79	AUC = 0.85
(70)	echo	Conventional	CL	HHD/HFePF	100	ACC = 0.81
(71)	echo	Conventional	CL/SVM	HFePF	397	AUC = 0.76
(72)	echo	Conventional	CL	HF	1,106	N/A

### 3.7. Abnormal Wall Motion

Most of the existing quantitative techniques for wall motion characterization involve laborious post-processing and image analysis. For this reason, ML approaches with a minimum user input and a correlation with the segmental cardiac function can improve clinical routine and triage.

For instance, Mantilla et al. (7) detected wall motion abnormalities in the left ventricle by means of spatiotemporal profiles obtained with pseudo delineations of 20 MRI patients. Wavelet and Fourier transforms were applied and the subsequent spaces were used to generate two models: SVM and dictionary learning (DICTL). Dictionary Learning at mid-cavity level obtained the best accuracy, 0.96 (Sp = Se = 0.96). Afshin et al. (73) exploited intensity distributions per segment. In their work, a reference frame automatically propagated to each cardiac phase generated the 16 segments for the whole cardiac cycle. LDA reduced feature dimensionality and linear SVM obtained an accuracy of 0.86 in a cohort of 58 MRI subjects.

Kusunose et al. (19) used a total of 300 patients with a history of myocardial infarction and 100 age-matched control patients. Each case contained echo from short-axis views at end-diastolic, mid-systolic, and end-systolic phases. An ensemble of 10 CNN models were trained. AUC obtained by the ML ensemble was similar to that produced by the cardiologists and sonographer readers (0.99 vs. 0.98, respectively), and the same occurred for territory detection (0.97 vs. 0.95, respectively). A summary of AWM studies can be found in Table 12.

**Table 12:** Selected studies using image-based ML analysis for diagnosis of wall motion abnormalities.

**Publication**	**Modality**	**Biomarker**	**ML technique**	**Diagnostic**	**Sample size**	**Performance**
(7)	MRI	Conventional	SVM/DICTL	AWM	20	ACC = 0.96
(73)	MRI	Conventional	SVM	AWM	58	ACC = 0.86
(19)	echo	Deep Learning	CNN	AWM	400	AUC = 0.99

## 4. Discussion and Future Perspectives

Reflected by the large amount of already published data reviewed above, AI in general and ML in particular have been shown to exhibit a huge potential to significantly influence diagnostic decision making in cardiology. In contrast to “traditional” statistical methods, the techniques from the field of AI are able to deal with large amounts of data (“big data”) and to integrate information from all fields of clinical care, including e.g., clinical parameters (“clinomics”), genetic information (“genomics”), protein metabolism (“proteomics”), and imaging data (“radiomics”) within one large all-encompassing analysis framework. The steadily increasing computational power and the increasing availability of data through mobile applications and the digital transformation of the global healthcare systems further contribute to the advancement of the field. Consequently, future studies will continue the use of these techniques in order to allow translation into routine clinical practice and thus pave the way toward improved diagnostic decision making tailored to individual patient-specific needs (subsumed under the heading “precision medicine”).

**Figure 8:**
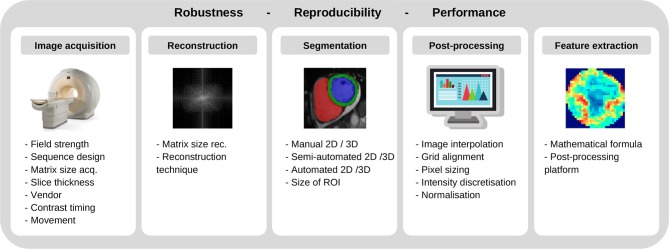
Factors involving robustness and reproducibility of quantitative imaging features.

Yet, in today's clinical routine, diagnostic decisions are still drawn from stand-alone parameters [e.g., LV ejection fraction, (74)], despite many encouraging research studies from the field of AI. On a per-patient basis, the diagnostic and prognostic value of such independent functional parameters was found to be low, Park and Kim (75). Given the diversity of cardiovascular imaging modalities, their potential additive value for more accurate diagnostics and risk stratification remains unclear. Besides, continued reliance on subjective visual interpretation, has resulted in considerable observer-dependencies and lack of standardization. The application of AI and precision medicine to CVD, however, is currently still is in its infancy, and faces huge challenges which have to be overcome by future research. To establish novel imaging biomarkers and AI techniques, the robustness and reproducibility of quantitative imaging features must be ensured, Zwanenburg et al. (76). Up to now, trained models and algorithms have limited generalizability due to the multiplicity of potential influencing factors (including differing scanners, vendors, CT radiation doses, MRI field strengths, sequences, sequence parameters, spatial and temporal resolutions, reconstruction algorithms, reconstruction parameters, and so forth; Figure 8).

For CT and positron emission tomography (PET) imaging, a variety of studies have highlighted difficulties in producing reliably reproducible radiomic features when using different vendors, scanners, and acquisition or reconstruction settings (48, 77–84). While the “image biomarker standardization initiative” (IBSI) has established certain standards for radiomic studies, Zwanenburg et al. (76), the specific needs of cardiac imaging have not yet been met. For cardiac CT, Hinzpeter et al. and Mannil et al. have investigated the influence of slice thickness, Hinzpeter et al. (84), and iterative reconstruction algorithms, Mannil et al. (48), on the robustness and comparability of radiomics features – observing considerable feature variations for differing technical settings. In contrast to this evolving body of literature on CT imaging, little evidence exists concerning the robustness of radiomic features in MRI (75, 85–87). Given the qualitative nature of most MRI sequences and the absence of absolute signal intensities (in contrast to CT imaging for instance), the robustness of radiomic features seems to heavily depend on acquisition sequences as well as acquisition and reconstruction parameters. In a recent phantom study, Baeßler et al. sought to evaluate the influence of different acquisition sequences, spatial resolution, and postprocessing settings (88) revealing that the robustness of radiomic features was heavily influenced by the acquisition sequence and image resolution as well as image processing settings. Future work not only needs to add to the understanding of such influencing factors but should also merge into extensive standardization efforts to ensure reliability of all imaging measures.

Several attempts to improve radiomic feature robustness through image normalization have been made. For more reliable quantification of emphysema, normalization was proposed for chest CT images reconstructed with different kernels, Gallardo-Estrella et al. (89). The proposed method decomposed each scan into multiple frequency bands, the energy of which was then normalized to the average energies observed in a set of scans reconstructed with a reference kernel. Building on these results, Jin et al. used a deep learning-based strategy for CT image normalization by means of a U-Net, Jin et al. (90). For harmonization of MRI images, similar deep learning algorithms were proposed for dynamic contrast enhanced (DCE) images in breast, Samala et al. (91), and brain MRI, Dewey et al. (92). Although yielding promising results, the applicability of such approaches in cardiovascular applications remains elusive, which is due to inherent particularities of cardiac imaging. Other than breast and brain, the human heart is steadily moving because of breathing and myocardial contraction. Second, the contrast bolus inside the ventricular lumen may influence the myocardial features. Aside from these specific characteristics, the impact of image normalization on extracted radiomic features has not been fully investigated yet. Besides lack of standardization of technical factors, the recent trend to train ML classifiers on relatively small datasets is a major issue of current methodology and hampers translation of the novel techniques into routine clinical practice. The small sample sizes in most cardiovascular imaging studies (usually N < 100 with > 1,000 variables in the models) lead to a considerable risk of overfitting. Overfitting leads to poor generalisability of the classification models when deployed to different datasets. Besides the current lack of imaging feature standardization and the problem of model-overfitting, other challenges should be acknowledged when it comes to translation of AI to daily patient care. While big data aims to integrate data from various sources, the current lack of interoperability of many systems used in clinical care poses huge obstacles for data pooling approaches. Several national and international attempts are currently under way to solve interoperability issues for medical care and to allow a seamless integration of different databases and informatic systems used in healthcare.

The ability to understand the rationale behind ML generated diagnostic grouping may be crucial in order to achieve widespread clinical use of this novel technology. However, especially with DL techniques, those are usually considered as being “black boxes,” which do not deliver any insights or explanations on how they reached their conclusions and upon which, e.g., imaging features, they based their decision. Although several attempts and ongoing research exist on delivering insights into an algorithm's decision making (such as heatmaps), these attempts are not sufficiently elaborated so far to convince most cardiology practitioners to use a diagnostic black box in daily clinical patient management. Thus, interpretability of DL models including the psychological aspects of digital transformation itself should represent one major aim of future research. Radiomics might represent a valid alternative for the meantime, since radiomic models—in cases where an appropriate and stepwise feature reduction is performed before training the ML algorithm—deliver more insights into the specific imaging features which were important for the model's classification performance. In summary, solutions achieving better standardization or normalization resulting in better generalisability are an important condition to bring radiomics and AI into cardiac precision medicine with concomitant improved diagnostic approaches to CVDs. In addition, better interoperability of healthcare informatics systems should be achieved. Finally, the steadfast progression of AI approaches to clinical decision making represent an abrupt change from conventional medical reasoning, as such, it is essential to engage with the psychological impact of the ongoing digital transformation in order to facilitate the transition of medical practice in line with advancing technologies. The extensive and encouraging work reviewed in this article above pursues one common goal for the future of cardiovascular medicine: to pave the way toward better diagnosis and precision medicine in cardiology. The application of AI to cardiology holds the promise to revolutionize individual disease monitoring and treatment (93), thus overcoming the currently used “one size fits all” approach derived from large clinical studies.

## Author Contributions

CM-I performed the literature search and its categorization, developed statistics, wrote the diagnostics literature section, and coordinated the remaining sections. VC wrote the machine learning section and contributed to the remaining sections. CI wrote the data section and designed the figures. KL supervised the manuscript development, wrote the abstract and the introduction, and reviewed all sections. ZR-E, BB, and SP contributed to drafting and critical appraisal of the manuscript. All authors reviewed the manuscript.

### Conflict of Interest

The authors declare that the research was conducted in the absence of any commercial or financial relationships that could be construed as a potential conflict of interest.

## Abbreviations

Machine learning abbreviations
AI: Articial Intelligence
AUC: Area Under Curve
ANN: Artificial Neural Networks
BN: Bayesian Network
CNN: Convolutional Neural Network
CL: Clustering
DL: Deep Learning
DT: Decision Tree
GA: Genetic Algorithm
GAN: Generative Adversarial Network
GBRT: Gradient Boosting Trees
kNN: k-Nearest Neighbors
LDA: Linear Discriminant Analysis
LR: Logistic Regression
ML: Machine Learning
PCA: Principal Component Analysis
PLS: Partial Least Squares
RF: Random Forest
ROC: Receiver Operating Characteristic Curve
Se: Sensitivity
Sp: Specificity
SVM: Support Vector Machine
VAE: Variational Autoencoder.
Cardiac imaging and clinical abbreviations
ARV: Abnormal Right Ventricle
ASD: Atrial Septal Defect
CAC: Coronary Artery Calcium
CAD: Coronary Artery Disease
CMR: Cardiac Magnetic Resonance
CT: Computed Tomography
CTA: Computed Tomography Angiography
CVD: Cardiovascular Disease
DCM: Dilated Cardiomyopathy
ECG: Electrocardiography
echo: Echocardiography
HCM: Hypertrophic Cardiomyopathy
HF: Heart Failure
HFpEF: Heart Failure with preserved Ejection Fraction
HHD: Hypertensive Heart Disease
ICA: Invasive Coronary Angiography
IR: Iterative Reconstruction
LV: Left Ventricle
MACE: Major Adverse Cardiovascular Event
MI: Myocardial Infarction
MR: Mitral Regurgitation
MRI: Magnetic Resonance Imaging
MYO: Myocarditis
NRS: Napkin Ring Sign
PET: Positron Emission Tomography
ROI: Region Of Interest
RV: Right Ventricle
SPECT: Single Positron Emission Computed Tomography.
